# Effect of Different Backgrounds and Instrumental Techniques on the Optical Properties and Shade Reproducibility of a Lithium Disilicate Glass–Ceramic

**DOI:** 10.1155/ijod/3208752

**Published:** 2026-11-04

**Authors:** Turki S. Alkhallagi

**Affiliations:** ^1^ Oral and Maxillofacial Prosthodontics Department Faculty of Dentistry King Abdulaziz University Jeddah Saudi Arabia kau.edu.sa

**Keywords:** color matching, digital shade matching, intraoral scanner, lithium disilicate glass–ceramic

## Abstract

**Objectives:**

During the shade‐matching step, digital shade‐detection tools can assist the dentist, but background factors may influence color measurement. The purpose of this study was to evaluate the influence of background on color measurements, to compare different digital shade‐detection methods, and to assess the reliability and consistency of these methods in color detection and shade matching for CAD/CAM lithium disilicate glass–ceramic.

**Materials and Methods:**

About 15 tile‐shaped ceramic IPS e.max CAD specimens were measured using various shade‐detection devices. VITA Easyshade Advance 5.0, Trios 3 and 5, and Medit i700 intraoral scanners were used to measure VITA Classical Shade and VITA 3D Master Shade on white, gray, and black backgrounds. Repeated measurements were done for each specimen. Separate linear mixed‐effects models were constructed for each shade system to analyze the effects of instrument, background, and time.

**Results:**

Shade measurements were significantly influenced by the instrument and background in both shade systems. For the VITA Classical Shade, instruments and backgrounds showed significant effects, with Medit exhibiting the greatest background‐related variation, while Trios 3 remained comparatively stable. White backgrounds produced higher VITA Classical Shade values than did gray or black backgrounds. For the VITA 3D Master Shade, instrument, background, and time were all significant, with VITA Easyshade Advance 5.0 showing large increases on black backgrounds and notable time‐related changes. Trios 3 and Trios 5 demonstrated greater stability across backgrounds and repeated measurements. Overall, Trios 3 provided the most consistent performance.

**Conclusions:**

The background conditions and instrumental techniques influenced the measured shade outcomes and shade reproducibility of the ceramic. The Trios 3 intraoral scanner achieved the highest consistency in VITA Classical Shade measurements across all backgrounds. A gray background showed the highest probability of matching manufacturer VITA Classical Shade designations.

**Clinical Significance:** Background can affect color measurements when using digital shade‐detection tools. Selecting the correct background and tool improves ceramic shade matching.

## 1. Introduction

Dental restorations should restore both function and esthetics [1, 2]. The desire to reproduce natural optical appearance in dental restorations and to meet the esthetic expectation of patients and dentists has led to the development of new restorative materials and instruments to determine and reproduce color [3].

Lithium disilicate is widely used for its combination of durability and esthetics [4–11]. Ceramic translucency, background color, cement thickness, and color were reported to significantly influence the color of lithium disilicate restorations [12–18].

To identify a tooth’s color and reproduce it in a dental restoration, commercial shade guides have been used [19].

The VITA Classical Shade guide (VITA Zahnfabrik, Bad Sackingen, Germany) is a widely used shade guide in dentistry. However, it cannot present hue, chroma, and value individually [19, 20]. The VITA 3D‐Master shade guides (VITA Zahnfabrik, Bad Sackingen, Germany) are also reported to increase chances of success in tooth shade matching due to the resemblance of human tooth shade and allow each dimension of color to be distinguished independently [19, 20]. VITA Easyshade Advance 5.0 can be used as an objective color measurement tool [21].

Digital cameras, spectrophotometers, and colorimeters have been used to assist in color determination and have been reported to be more reliable than the visual method [3, 22, 23]. They can also be used to determine the translucency of the tooth, which is the second leading factor, after color, for matching a restoration to adjacent teeth [24].

Intraoral digital scanners (Trios 3 and 5; 3Shape, Dentsply DeguDent, Medit i700, and others) have been increasingly used to capture digital dental arch scans and provide shade‐taking functionality [25]. This feature may lead to greater convenience and enhanced lab communication. However, whether an intraoral digital scanner with an integrated shade‐taking function is accurate and reproducible remains unclear [26–29].

In dentistry, color is commonly described using hue, value, and chroma according to the Munsell color system [30–32]. The *L* ^∗^, *a* ^∗^, *b* ^∗^ color space, also known as CIEL ^∗^ *a* ^∗^ *b* ^∗^, is another commonly used system for shade matching [30, 32]. The delta *E* (Δ*E*) metric, used for assessing accuracy, serves as a numerical gauge for the color distinction between two objects, facilitating comparative analysis. As Δ*E* increases, the object shows greater color dissimilarity [32, 33]. According to previous studies, the human eye cannot distinguish between different colors in a clinical setting until the Δ*E* value reaches 3.3 [34, 35]. This underscores the necessity of using advanced technologies, especially in restoring anterior esthetic regions.

The purpose of the present study was to evaluate the influence of background on color measurements, to compare different digital shade‐detection methods, and to assess the reliability and consistency of these methods in color detection and shade matching for CAD/CAM lithium disilicate glass–ceramic.

### 1.1. Null Hypothesis (*H*_0_)

There is no significant difference across all backgrounds or machines in assessing the consistency and reliability of shade detection.

### 1.2. Alternative Hypothesis (*H*_1_)

There would be a significant difference between all backgrounds and the machines used in assessing the consistency and reliability of shade detection.

## 2. Materials and Methods

### 2.1. Specimen Preparation

A total of 15 ceramic specimens in tile shape with 2 mm thickness were obtained from a lithium disilicate glass–ceramic (IPS e.max CAD high translucency (HT)/A2, Ivoclar Vivadent, Schaan, Liechtenstein). Three blocks of IPS e.max CAD were sectioned using a precision saw machine (ISOMET5000, Buehler, Lake Bluff, IL). Both sides of each specimen were finished to a uniform surface with diamond abrasive polishing disks using the EcoMet polishing and grinding machine (Buehler, EcoMet. 250 Grinder‐Polisher, Product Number: 497250). The final thickness of 2 mm was inspected with a digital micrometer (Quantumike 0–25 mm/0.001 mm, Mitutoyo Corporation, Kawasaki, Kanagawa, Japan).

### 2.2. Specimen Treatment

The specimens were subjected to heat treatment following the manufacturer’s instructions to promote final crystallization (initial temperature 403°C, heating rate 1–90°C/min up to 820°C, hold time 10 s, heating rate 2–30°C/min up to 840°C, hold time 7 min, long‐term cooling at 700°C; vacuum 1 at 550 until 820°C, and vacuum 2 at 820–840°C) using the Programat CS furnace (Ivoclar Vivadent, Schaan, Liechtenstein). The specimens were allowed to cool to room temperature after the process.

### 2.3. Specimen Measurement

Fifteen specimens were measured using different shade‐detection devices. The portable digital spectrophotometer (VITA Easyshade Advance 5.0, VITA Zahnfabrik, Bad Sackingen, Germany) and three intraoral scanners: 3shape (Trios 3 and 5) (3Shape, Copenhagen, Denmark) and Medit i700 (Medit Crop, Seoul, Korea) were used to measure VITA Classical Shade, and VITA 3D Master Shade. Each specimen was measured three consecutive times, with instrument repositioning between measurements, over three backgrounds: white, gray, and black. Before each measurement, the device was calibrated, as indicated by the manufacturer.

All measurements were made at the center of the specimen and performed by a single trained operator under standardized D65 illumination. A total of 540 measurements per shade system were recorded and saved in an Excel sheet.

The spectrophotometer used in this study was VITA Easyshade Advance 5.0 (VITA Easyshade Advance 5.0, VITA Zahnfabrik, Bad Sackingen, Germany). After the instrument calibration, following the manufacturer’s recommendations, the instrument was placed on the specimen at the same angle (90°) at the middle of the specimen, and then, the shade assessment was automatically generated on the screen. The shade was measured three times for each background for a total of 9 measurements per specimen. Shade measurement and color coordinates of all specimens were performed in the “Tooth Single” mode to determine the shade in both VITA Classical Shade and VITA 3D Master Shade systems.

The 3Shape Trios 3 and 5 (3Shape, Copenhagen, Denmark) and the Medit i700 intraoral scanners (Medit Crop, Seoul, Korea) were used in shade detection. Automatic calibration was performed on all devices according to the manufacturer’s instructions. The shade assessment was generated automatically when the examiner selected the shade detection feature and placed the shade measurement circle at the nearest point to the center of each specimen. This point should show the results for both shade guide systems (VITA Classical Shade and VITA 3D Master Shade) results. Any points not shown in both systems were ignored, and another measurement was taken. Three separate scans were performed for each specimen under each background condition. For each scan, the scanner was repositioned at the center of the specimen before the subsequent scan was initiated. Each scan was processed by the scanner software to determine the specimen shade in the VITA Classical Shade and VITA 3D Master Shade systems. Thus, the three repeated measurements represented three separate scan acquisitions rather than repeated analyses of a single captured image. Scanner software was used to determine the shade in VITA Classical Shade and VITA 3D Master Shade systems.

### 2.4. Statistical Analysis

A formal sample size calculation (power analysis) was performed using 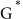
Power (Version 3.1), based on a conventional medium effect size (Cohen’s *f* = 0.25), *α* = 0.05, and statistical power of 0.80, to determine the minimum required number of specimens.

Data were imported into SPSS Statistics v30 (IBM SPSS Inc., Armonk, NY). Fifteen specimens were tested in this study (*n* = 15).

Each specimen was evaluated under 12 experimental conditions defined by the combination of four shade‐measuring instruments (VITA Easyshade Advance 5.0, Medit i700, Trios 3, and Trios 5) and three background conditions (white, gray, and black), resulting in 180 specimen–instrument–background combinations per shade system. Each condition was measured three times, resulting in a total of 540 measurements per shade system (1080 measurements in total for both shade systems). All repeated measurements were retained as individual observations and were not averaged prior to the statistical analysis.

Two dependent variables were analyzed separately: the VITA Classical Shade score and the VITA 3D Master Shade score.

The primary statistical analysis was a linear mixed‐effects model used to evaluate the effects of instrument type, background, and repeated measurements (time), as well as their interactions.

To permit parametric analysis, the categorical shade outcomes were converted to numeric ranks according to their predefined sequential order within each respective shade guide system before modeling. Each distinct shade designation was assigned a consecutive numeric rank, with the same coding scheme being applied consistently across all instruments and background conditions. The numeric values represented the ordinal position of the reported shade designation and were not interpreted as direct colorimetric measurements. Because repeated measurements were obtained from the same specimen, separate linear mixed‐effects models were constructed for the VITA Classical Shade and VITA 3D Master Shade outcomes. In each model, instrument, background, and time (measurements 1, 2, and 3) were entered as fixed effects. The instrument×background and instrument×time interaction terms were also included. A random intercept for the specimen was specified to account for between‐specimen variation, and the repeated measurements across time were modeled with an unstructured covariance matrix.

All inferential statistics were derived from the linear mixed‐effects models as the primary analytical framework.

Models were estimated with restricted maximum likelihood, degrees of freedom were approximated with the Satterthwaite method, and pairwise comparisons of estimated marginal means were adjusted by the Bonferroni procedure. The significance level was set at *α* = 0.05.

## 3. Results

For the VITA Classical Shade, significant main effects were found for instrument (*F* = 37.179; *p*  < 0.001) and background (*F* = 31.513; *p*  < 0.001), whereas the main effect of time was not significant (*F* = 2.550; *p* = 0.081) as shown in Table 1. The instrument × background interaction was significant (*F* = 32.137; *p*  < 0.001), but the instrument × time interaction was not (*F* = 0.916; *p* = 0.485).

**Table 1:** Univariate tests for main effects on VITA Classical Shade.

Effect	df1	df2	*F*	*p*
Instrument	3	164.364	37.179	<0.001
Background	2	168.151	31.513	<0.001

*Note*: Dependent variable = VITA Classical Shade. *F* tests are based on the linearly independent pairwise comparisons among the estimated marginal means. Significant main effects were observed for both instrument and background on VITA Classical Shade outcomes (*p*  < 0.001), indicating that shade measurements varied significantly according to device type and background condition.

The estimated marginal means for VITA Classical Shade were 1.052 for VITA Easyshade Advance 5.0, 2.496 for Medit, 1.933 for Trios 3, and 1.978 for Trios 5, as shown in Table 2. VITA Easyshade Advance 5.0 yielded significantly lower scores than Medit, Trios 3, and Trios 5 (all *p*  < 0.001). Medit yielded significantly higher scores than Trios 3 (*p*  < 0.001) and Trios 5 (*p* = 0.002), whereas Trios 3 and Trios 5 did not differ significantly (*p* = 1.000). Table 3 shows the estimated marginal means for VITA Classical Shade across backgrounds, which were 2.382 for white, 1.628 for gray, and 1.584 for black. White background produced significantly higher VITA Classical Shade scores than gray and black (both *p*  < 0.001), whereas gray and black did not differ (*p* = 1.000).

**Table 2:** The estimated marginal means for VITA Classical Shade for all instruments.

Instrument	Mean	Std. error	df	95% Confidence interval	
				Lower bound	Upper bound
VITA Easyshade Advance 5.0	1.052	0.098	164.364	0.858	1.246
Medit	2.496	0.098	164.364	2.302	2.690
Trios 3	1.933	0.098	164.364	1.739	2.127
Trios 5	1.978	0.098	164.364	1.784	2.172

*Note:* Estimated marginal means showed differences among instruments in VITA Classical Shade measurements, with Medit demonstrating the highest mean values and VITA Easyshade Advance 5.0 the lowest, indicating variability in shade readings across devices. Dependent variable: VITA Classical Shade.

**Table 3:** The estimated marginal means for VITA Classical Shade across all backgrounds.

Background	Mean	Std. error	df	95% Confidence interval	
				Lower bound	Upper bound
White	2.382	0.082	177.237	2.221	2.544
Gray	1.628	0.082	177.237	1.466	1.789
Black	1.584	0.082	177.237	1.423	1.746

*Note:* Significant differences were observed across background conditions for VITA Classical Shade measurements. The white background produced higher estimated marginal means compared with gray and black backgrounds. Dependent variable: VITA Classical Shade.

The significant instrument × background interaction for VITA Classical Shade indicated that the magnitude and direction of background‐related change differed among instruments. The most pronounced deviation was observed for Medit, which showed a markedly higher mean on the white background (4.515) than that on gray (1.822) or black (1.152). VITA Easyshade Advance 5.0 remained essentially unchanged across backgrounds (1.050–1.056), whereas Trios 3 showed only small differences (1.826–2.062). Trios 5 exhibited a moderate increase on the black background (2.303) relative to white (1.903) and gray (1.728), as shown in Tables 4 and 5.

**Table 4:** The instrument × background interaction for VITA Classical Shade.

Instrument	Background	Mean	Std. error	df	95% Confidence interval	
					Lower bound	Upper bound
VITA Easyshade Advance 5.0	White	1.050	0.163	177.237	0.727	1.372
	Gray	1.050	0.163	177.237	0.727	1.372
	Black	1.056	0.163	177.237	0.734	1.379
Medit	White	4.515	0.163	177.237	4.193	4.838
	Gray	1.822	0.163	177.237	1.499	2.144
	Black	1.152	0.163	177.237	0.830	1.474
Trios 3	White	2.062	0.163	177.237	1.740	2.385
	Gray	1.911	0.163	177.237	1.589	2.234
	Black	1.826	0.163	177.237	1.504	2.149
Trios 5	White	1.903	0.163	177.237	1.580	2.225
	Gray	1.728	0.163	177.237	1.405	2.050
	Black	2.303	0.163	177.237	1.981	2.626

*Note:* The effect of background on VITA Classical Shade measurements differed among instruments. Medit showed the greatest variation across backgrounds, whereas VITA Easyshade Advance 5.0 remained relatively stable and Trios 3 demonstrated only minor changes. Dependent variable: VITA Classical Shade.

**Table 5:** The instrument × time interaction for VITA Classical Shade.

Instrument	Time	Mean	Std. error	df	95% Confidence interval	
					Lower bound	Upper bound
VITA Easyshade Advance 5.0	1	1.000	0.117	168.848	0.770	1.230
	2	1.067	0.133	171.112	0.804	1.329
	3	1.089	0.147	160.582	0.799	1.379
Medit	1	2.422	0.117	168.848	2.192	2.653
	2	2.644	0.133	171.112	2.382	2.907
	3	2.422	0.147	160.582	2.132	2.712
Trios 3	1	1.844	0.117	168.848	1.614	2.075
	2	1.978	0.133	171.112	1.715	2.240
	3	1.978	0.147	160.582	1.688	2.268
Trios 5	1	1.756	0.117	168.848	1.525	1.986
	2	2.022	0.133	171.112	1.760	2.285
	3	2.156	0.147	160.582	1.865	2.446

*Note:* No significant instrument × time interaction was observed for VITA Classical Shade measurements, indicating that repeated measurements produced consistent results across all instruments. Dependent variable: VITA Classical Shade.

For the VITA 3D Master Shade, significant main effects were found for instrument (*F* = 100.494; *p*  < 0.001), background (*F* = 65.196; *p*  < 0.001), and time (*F* = 5.767; *p* = 0.004). Both the instrument × background interaction (*F* = 82.458; *p*  < 0.001) and the instrument × time interaction (*F* = 8.667; *p*  < 0.001) were also significant, as shown in Table 6.

**Table 6:** Univariate tests for main effects on VITA 3D Master Shade.

Effect	df1	df2	*F*	*p*
Instrument	3	79.659	100.494	<0.001
Background	2	166.294	65.196	<0.001

*Note:* Dependent variable = VITA 3D Master Shade. *F* tests are based on the linearly independent pairwise comparisons among the estimated marginal means. Significant main effects of instrument and background were observed for VITA 3D Master Shade measurements, indicating that both the shade‐measuring device and the background condition significantly influenced the recorded shade values.

The estimated marginal means for VITA 3D Master Shade were 6.756 for VITA Easyshade Advance 5.0, 4.630 for Medit, 4.970 for Trios 3, and 4.563 for Trios 5, as shown in Table 7. VITA Easyshade Advance 5.0 produced significantly higher scores than Medit, Trios 3, and Trios 5 (all *p*  < 0.001). Trios 3 was also slightly higher than Trios 5 (*p* = 0.039), whereas the Medit‐Trios 3 (*p* = 0.131) and Medit‐Trios 5 (*p* = 1.000) comparisons were not significant. Table 8 shows the estimated marginal means for VITA 3D Master Shade across backgrounds, which were 4.916 for white, 4.924 for gray, and 5.849 for black. Black background produced significantly higher VITA 3D Master Shade scores than white and gray (both *p*  < 0.001), whereas white and gray did not differ (*p* = 1.000).

**Table 7:** The estimated marginal means for VITA 3D Master Shade for all instruments.

Instrument	Mean	Std. error	df	95% Confidence interval	
				Lower bound	Upper bound
VITA Easyshade Advance 5.0	6.756	0.103	79.659	6.551	6.961
Medit	4.630	0.103	79.659	4.425	4.835
Trios 3	4.970	0.103	79.659	4.765	5.175
Trios 5	4.563	0.103	79.659	4.358	4.768

*Note:* Estimated marginal means differed among the evaluated instruments for VITA 3D Master Shade measurements, indicating variability in shade determination between the digital shade‐detection devices. Dependent variable: VITA 3D Master Shade.

**Table 8:** The estimated marginal means for VITA 3D Master Shade across all backgrounds.

Background	Mean	Std. error	df	95% Confidence interval	
				Lower bound	Upper bound
White	4.916	0.075	163.675	4.769	5.064
Gray	4.924	0.075	163.675	4.776	5.072
Black	5.849	0.075	163.675	5.701	5.996

*Note:* Background significantly influenced VITA 3D Master Shade measurements. The black background yielded higher estimated marginal means than the white and gray backgrounds, while the latter two did not differ significantly. Dependent variable: VITA 3D Master Shade.

The significant interactions in the VITA 3D Master Shade showed that both background and repeated measurement affected instruments differently, as shown in Tables 9 and 10. For background, VITA Easyshade Advance 5.0 had substantially higher scores on black (9.346) than on white or gray (both 5.460), while Medit showed the opposite pattern, with its lowest value on black (4.088). Trios 3 was comparatively stable across backgrounds (4.848–5.035), and Trios 5 increased gradually from white (4.061) to gray (4.696) to black (4.932). Across repeated measurements, VITA Easyshade Advance 5.0 decreased from 8.000 at the first measurement to 6.133 at the second and third measurements, whereas Medit, Trios 3, and Trios 5 demonstrated only minimal time‐related changes.

**Table 9:** The instrument × background interaction for VITA 3D Master Shade.

Instrument	Background	Mean	Std. error	df	95% Confidence interval	
					Lower bound	Upper bound
VITA Easyshade Advance 5.0	White	5.460	0.150	163.675	5.165	5.756
	Gray	5.460	0.150	163.675	5.165	5.756
	Black	9.346	0.150	163.675	9.051	9.641
Medit	White	5.108	0.150	163.675	4.813	5.404
	Gray	4.692	0.150	163.675	4.397	4.987
	Black	4.088	0.150	163.675	3.793	4.384
Trios 3	White	5.035	0.150	163.675	4.740	5.331
	Gray	4.848	0.150	163.675	4.553	5.143
	Black	5.028	0.150	163.675	4.733	5.323
Trios 5	White	4.061	0.150	163.675	3.766	4.356
	Gray	4.696	0.150	163.675	4.401	4.991
	Black	4.932	0.150	163.675	4.637	5.227

*Note:* The effect of background on VITA 3D Master Shade measurements differed among instruments. VITA Easyshade Advance 5.0 showed the greatest variation across backgrounds, whereas Trios 3 demonstrated only minor changes. Dependent variable: VITA 3D Master Shade.

**Table 10:** The instrument × time interaction for VITA 3D Master Shade.

Instrument	Time	Mean	Std. error	df	95% Confidence interval	
					Lower bound	Upper bound
VITA Easyshade Advance 5.0	1	8.000	0.118	78.753	7.765	8.235
	2	6.133	0.170	100.547	5.796	6.471
	3	6.133	0.177	107.587	5.783	6.484
Medit	1	4.667	0.118	78.753	4.431	4.902
	2	4.600	0.170	100.547	4.263	4.937
	3	4.622	0.177	107.587	4.272	4.973
Trios 3	1	4.889	0.118	78.753	4.654	5.124
	2	5.022	0.170	100.547	4.685	5.360
	3	5.000	0.177	107.587	4.650	5.350
Trios 5	1	4.422	0.118	78.753	4.187	4.657
	2	4.600	0.170	100.547	4.263	4.937
	3	4.667	0.177	107.587	4.316	5.017

*Note:* The effect of time on VITA 3D Master Shade measurements differed among instruments. VITA Easyshade Advance 5.0 showed the greatest variation, whereas Medit and Trios 5 demonstrated only minor changes. Dependent variable: VITA 3D Master Shade.

Taken together, the findings indicate that shade detection was influenced not only by the instrument used but also by the optical background and that this influence depended on the shade system being analyzed. For VITA Classical Shade, instrument differences were clear, and background effects were present, but the repeated measurement itself did not materially change the scores. This pattern suggests that, within the VITA Classical Shade system, the principal source of variation was the combination of device characteristics and background reflectance rather than short‐term measurement repeatability.

The VITA 3D Master Shade outcome was more sensitive than the VITA Classical Shade outcome to both background and repeated measurement. The significant time effect and instrument×time interaction were driven mainly by VITA Easyshade Advance 5.0, whose first measurement was substantially higher than subsequent measurements. This pattern may reflect instrument‐specific settling, calibration, or measurement behavior rather than a true change in the specimen shade. In contrast, Medit, Trios 3, and Trios 5 showed greater temporal stability across the 3 repeated measurements.

Clinically, the interaction findings are more important than the main effects alone. The white background inflated VITA Classical Shade scores primarily for Medit, whereas the black background inflated VITA 3D Master Shade scores primarily for VITA Easyshade Advance 5.0. Therefore, direct comparison of shade values across instruments should be made with caution unless background conditions are standardized. Instruments that appeared relatively stable across backgrounds, especially Trios 3 and to a lesser extent Trios 5, may provide more robust performance when background control is imperfect.

## 4. Discussion

The null hypothesis was rejected; the background and the machines used significantly affect shade consistency and the reliability of shade‐detection. A statistically significant difference in shade measurements for the VITA Classical Shade was observed across all backgrounds and devices used. The highest probability of detecting manufacturer‐defined VITA Classical Shade was observed when a gray background was used, which is supporting previous studies that suggest neutral backgrounds are most reliable for shade matching [36]. The white background had less consistent results with all digital devices, which may have influenced the shade measurement. Therefore, selecting the appropriate background is important when performing shade assessments.

The shade‐detection devices showed different levels of consistency as Trios 3 appeared relatively stable across all backgrounds in both VITA Classical Shade and VITA 3D Master Shade systems. On the other hand, VITA Easyshade Advance 5.0 and Medit i700 showed less reliable results. The Trios 3 consistently achieved results closest to the manufacturer’s VITA Classical Shade, similar to Reyes et al.’s [19] study. These results align with previous studies that have highlighted the superior accuracy of the Trios 3 intraoral scanner compared to other intraoral scanners in clinical practice [21]. The performance discrepancy between devices on gray and black backgrounds suggests that some devices may be more sensitive to background color or lighting conditions, limiting their clinical applicability in certain scenarios [30].

Although the samples of the present study had different thickness compared to previous studies, the present study had the same results as previous studies [37]. The gray background had the highest probability of detecting the manufacturer‐defined VITA Classical Shade. The improved stability observed with gray backgrounds may be explained by their neutral reflectance properties, which reduce color bias and minimize light reflection variability compared with those of white or black surfaces. White backgrounds may introduce excessive light reflection, potentially increasing perceived brightness, while black backgrounds may absorb light and reduce signal stability, both of which can affect digital shade interpretation. In contrast, gray surfaces provide an intermediate reflectance level that may support more balanced optical detection across different devices.

From a clinical perspective, standardization of background conditions during shade selection may be achieved by using neutral gray intraoral or extraoral backgrounds, such as gray shade tabs, neutral photographic backdrops, or controlled clinical shade‐matching environments with neutral‐colored drapes and reduced environmental color contamination. Although complete standardization in daily practice is challenging, minimizing strong contrasting colors in the visual field may improve shade consistency.

The present study was designed to evaluate the influence of different backgrounds and instrumental techniques on the optical properties and shade reproducibility of a lithium disilicate glass–ceramic manufactured using CAD‐CAM technology, with the manufacturer VITA Classical Shade used as the control. The study results showed that background and digital device selection affect the reproducibility of shade detection. These findings are important in the clinical setting as they can guide practitioners to select the appropriate background and digital device to achieve optimal shade matching for lithium disilicate glass–ceramic.

Shade detection is a critical factor in the esthetic success of dental restorations [1, 2]. The data suggest that intraoral scanners, particularly Trios 3, offer reliable and reproducible shade‐detection ability and achieve consistent results [19, 21]. Digital devices could help dental practitioners improve patient esthetic outcomes, particularly in anterior restorations. Additionally, the study’s findings support previous studies that using neutral‐toned backgrounds may improve shade detection and reduce color mismatch [38].

This present study has several limitations that may restrict the generalizability of the findings. The sample size may not fully represent the range of shade and material variations seen in dental clinics as only one shade (HT/A2) with a specific thickness (2 mm) was tested. Future studies should consider including a more diverse set of materials and shades. Furthermore, while this study focused on the consistency of digital devices under controlled laboratory conditions, it is not similar to clinical settings. Patient lips, multicolor teeth, different light sources, ambient colors, and operator technique, all these variables could influence shade detection. Another limitation is that quantitative Δ*E*‐based colorimetric analysis was not performed. Therefore, direct comparison to clinically established perceptibility (Δ*E* ≈ 1–3) and acceptability (Δ*E* ≈ 3.3) thresholds is not possible. Nevertheless, the categorical shade‐matching outcomes observed across different devices and backgrounds provide a practical, clinically relevant assessment of shade reproducibility. Future studies incorporating Δ*E* measurements and testing under more realistic clinical conditions would allow a more precise and comprehensive evaluation of how background color, lighting, and operator factors affect shade detection.

These results support the conclusion that background standardization is essential in shade‐matching studies and that the choice of the instrument can substantially influence the recorded shade designation.

## 5. Conclusion

The backgrounds and digital devices influenced the optical property and shade reproducibility of the lithium disilicate glass–ceramic. Thus, within the limitations of the present study, the following conclusions can be drawn:

– The Trios 3 intraoral scanner showed the most consistent performance across different backgrounds and shade systems compared with the other tested devices.
– The gray background shows the greatest probability of detecting the manufacturer‐defined VITA Classical Shade.

## Funding

The author has nothing to report.

## Disclosure

The author does not have any financial interest in the companies whose materials are included in this article.

## Ethics Statement

This article does not contain any studies with human participants or animals performed by the author.

## Consent

For this type of study, formal consent is not required.

## Conflicts of Interest

The author declares no conflicts of interest.

## Data Availability

The data that support the findings of this study are available from the corresponding author upon reasonable request.

## References

[1] Shillingburg H. T. , Fundamentals of Fixed Prosthodontics, 1997, 3rd edition, Quintessence Pub. Co. ix.

[2] Rosenstiel S. F. , Land M. F. , and Fujimoto J. , Contemporary Fixed Prosthodontics, 2006, 4th edition, Mosby/Elsevier.

[3] Chen H. , Huang J. , and Dong X. , et al.A Systematic Review of Visual and Instrumental Measurements for Tooth Shade Matching, Quintessence International. (2012) 43, no. 8, 649–659. 23034418

[4] Bindl A. , Richter B. , and Mörmann W. H. , Survival of Ceramic Computer-Aided Design/Manufacturing Crowns Bonded to Preparations With Reduced Macroretention Geometry, International Journal of Prosthodontics. (2005) 18, no. 3, 219–224. 15945309

[5] Miyazaki T. , Hotta Y. , Kunii J. , Kuriyama S. , and Tamaki Y. , A Review of Dental CAD/CAM: Current Status and Future Perspectives From 20 Years of Experience, Dental Materials Journal. (2009) 28, no. 1, 44–56, 10.4012/dmj.28.44. 19280967

[6] Sen N. and Us Y. O. , Mechanical and Optical Properties of Monolithic CAD-CAM Restorative Materials, Journal of Prosthetic Dentistry. (2018) 119, no. 4, 593–599, 10.1016/j.prosdent.2017.06.012. 28781072

[7] Rosentritt M. , Preis V. , Behr M. , and Hahnel S. , Influence of Preparation, Fitting, and Cementation on the Vitro Performance and Fracture Resistance of CAD/CAM Crowns, Journal of Dentistry. (2017) 65, 70–75, 10.1016/j.jdent.2017.07.006. 28734997

[8] Schestatsky R. , Zucuni C. P. , and Venturini A. B. , et al.CAD-CAM Milled Versus Pressed Lithium-Disilicate Monolithic Crowns Adhesively Cemented After Distinct Surface Treatments: Fatigue Performance and Ceramic Surface Characteristics, Journal of the Mechanical Behavior of Biomedical Materials. (2019) 94, 144–154, 10.1016/j.jmbbm.2019.03.005. 30897502

[9] Schestatsky R. , Zucuni C. P. , and Dapieve K. S. , et al.Microstructure, Topography, Surface Roughness, Fractal Dimension, Internal and Marginal Adaptation of Pressed and Milled Lithium-Disilicate Monolithic Restorations, Journal of Prosthodontic Research. (2020) 64, no. 1, 12–19, 10.1016/j.jpor.2019.05.004. 31213364

[10] Poggio C. E. , Ercoli C. , Rispoli L. , Maiorana C. , Esposito M. , and Cochrane Oral Health Group , Metal-Free Materials for Fixed Prosthodontic Restorations, Cochrane Database of Systematic Reviews. (2017) 12, no. 12, 10.1002/14651858.CD009606.pub2, CD009606. PMC6486204 29261853

[11] Sailer I. , Makarov N. A. , Thoma D. S. , Zwahlen M. , and Pjetursson B. E. , All-Ceramic or Metal-Ceramic Tooth-Supported Fixed Dental Prostheses (FDPs)? A Systematic Review of the Survival and Complication Rates. Part I: Single Crowns (SCs), Dental Materials. (2015) 31, no. 6, 603–623. 10.1016/j.dental.2015.02.011 25842099

[12] İşeri U. , Özkurt Z. , Yalnız A. , and Kazazoğlu E. , Comparison of Different Grinding Procedures on the Flexural Strength of Zirconia, Journal of Prosthetic Dentistry. (2012) 107, no. 5, 309–315, 10.1016/S0022-3913(12)60081-X. 22546308

[13] Pereira G. K. R. , Fraga S. , Montagner A. F. , Soares F. Z. M. , Kleverlaan C. J. , and Valandro L. F. , The Effect of Grinding on the Mechanical Behavior of Y-TZP Ceramics: A Systematic Review and Meta-Analyses, Journal of the Mechanical Behavior of Biomedical Materials. (2016) 63, 417–442, 10.1016/j.jmbbm.2016.06.028. 27469603

[14] Zucuni C. P. , Guilardi L. D. F. , Rippe M. P. , Pereira G. K. R. , and Valandro L. F. , Fatigue Strength of Yttria-Stabilized Zirconia Polycrystals: Effects of Grinding, Polishing, Glazing, and Heat Treatment, Journal of the Mechanical Behavior of Biomedical Materials. (2017) 75, 512–520, 10.1016/j.jmbbm.2017.06.016. 28843883

[15] Alp G. , Subasi M. G. , Johnston W. M. , and Yilmaz B. , Effect of Surface Treatments and Coffee Thermocycling on the Color and Translucency of CAD-CAM Monolithic Glass-Ceramic, Journal of Prosthetic Dentistry. (2018) 120, no. 2, 263–268, 10.1016/j.prosdent.2017.10.024. 29551378

[16] Maciel L. C. , Silva C. F. B. , de Jesus R. H. , Concílio L. D. S. , Kano S. C. , and Xible A. A. , Influence of Polishing Systems on Roughness and Color Change of Two Dental Ceramics, Journal of Advanced Prosthodontics. (2019) 11, no. 4, 215–222, 10.4047/jap.2019.11.4.215. PMC6718845 31497269

[17] Green D. J. , A Technique for Introducing Surface Compression Into Zirconia Ceramics, Journal of the American Ceramic Society. (2006) 66, no. 10.

[18] Kosmač T. , Oblak C. , Jevnikar P. , Funduk N. , and Marion L. , The Effect of Surface Grinding and Sandblasting on Flexural Strength and Reliability of Y-TZP Zirconia Ceramic, Dental Materials. (1999) 15, no. 6, 426–433, 10.1016/S0109-5641(99)00070-6. 10863444

[19] Reyes J. , Acosta P. , and Ventura D. , Repeatability of the Human Eye Compared to an Intraoral Scanner in Dental Shade Matching, Heliyon. (2019) 5, no. 7, 10.1016/j.heliyon.2019.e02100, e02100. PMC6660562 31372552

[20] Sirintawat N. , Leelaratrungruang T. , Poovarodom P. , Kiattavorncharoen S. , and Amornsettachai P. , The Accuracy and Reliability of Tooth Shade Selection Using Different Instrumental Techniques: An In Vitro Study, Sensors. (2021) 21, no. 22, 10.3390/s21227490, 7490. PMC8620419 34833565

[21] Ebeid K. , Sabet A. , and Della Bona A. , Accuracy and Repeatability of Different Intraoral Scanners on Shade Determination, Journal of Esthetic and Restorative Dentistry. (2021) 33, no. 6, 844–848, 10.1111/jerd.12687. 33227179

[22] Schropp L. , Shade Matching Assisted by Digital Photography and Computer Software, Journal of Prosthodontics-Implant Esthetic and Reconstructive Dentistry. (2009) 18, no. 3, 235–241, 10.1111/j.1532-849X.2008.00409.x. 19141046

[23] Okubo S. R. , Kanawati A. , Richards M. W. , and Childressd S. , Evaluation of Visual and Instrument Shade Matching, Journal of Prosthetic Dentistry. (1998) 80, no. 6, 642–648, 10.1016/S0022-3913(98)70049-6. 9830067

[24] Lee Y.-K. , Translucency of Human Teeth and Dental Restorative Materials and Its Clinical Relevance, Journal of Biomedical Optics. (2015) 20, no. 4, 10.1117/1.JBO.20.4.045002, 045002. 25875626

[25] Tabatabaian F. , Beyabanaki E. , Alirezaei P. , and Epakchi S. , Visual and Digital Tooth Shade Selection Methods, Related Effective Factors and Conditions, and Their Accuracy and Precision: A Literature Review, Journal of Esthetic and Restorative Dentistry. (2021) 33, no. 8, 1084–1104, 10.1111/jerd.12816. 34498789

[26] Brandt J. , Nelson S. , Lauer H.-C. , von Hehn U. , and Brandt S. , In Vivo Study for Tooth Colour Determination—Visual Versus Digital, Clinical Oral Investigations. (2017) 21, no. 9, 2863–2871, 10.1007/s00784-017-2088-0. 28265772

[27] K G. , Effectiveness of Shade Measurements Using a Scanning and Computer Software System: A Pilot Study, International Journal of Oral and Dental Health. (2015) 1, no. 2.

[28] Amornvit P. , Rokaya D. , Peampring C. , and Sanohkan S. , Confocal 3D Optical Intraoral Scanners and Comparison of Image Capturing Accuracy, Computers, Materials & Continua. (2020) 66, no. 1, 303–314, 10.32604/cmc.2020.011943.

[29] Mehl A. , Bosch G. , Fischer C. , and Ender A. , In Vivo Tooth-Color Measurement With a New 3D Intraoral Scanning System in Comparison to Conventional Digital and Visual Color Determination Methods, International Journal of Computerized Dentistry. (2017) 20, no. 4, 343–361. 29292410

[30] Della Bona A. , Color and Appearance in Dentistry, 2020.

[31] Baltzer A. and Kaufmann-Jinoian V. , Shading of Ceramic Crowns Using Digital Tooth Shade Matching Devices, International Journal of Computerized Dentistry. (2005) 8, no. 2, 129–152. 16201397

[32] Vohra M. and Shenoy A. , In Vitro Evaluation of Accuracy and Reliability of Tooth Shade Selection Using Different Digital Instruments, Cureus. (2024) 16, no. 1, 10.7759/cureus.52363, e52363. PMC10868152 38361673

[33] Khashayar G. , Bain P. A. , Salari S. , Dozic A. , Kleverlaan C. J. , and Feilzer A. J. , Perceptibility and Acceptability Thresholds for Colour Differences in Dentistry, Journal of Dentistry. (2014) 42, no. 6, 637–644, 10.1016/j.jdent.2013.11.017. 24334221

[34] Miller L. L. , Shade Selection, Journal of Esthetic and Restorative Dentistry. (1994) 6, no. 2, 47–60. 7993674

[35] Carney M. N. and Johnston W. M. , The Development of a Novel Shade Selection Program for Fixed Shade Translucent Dental Materials, Journal of Dentistry. (2017) 62, 81–84, 10.1016/j.jdent.2017.05.002. PMC5560996 28526444

[36] Pérez M. D. , Della Bona A. , Carrillo-Pérez F. , Dudea D. , Pecho O. E. , and Herrera L. J. , Does Background Color Influence Visual Thresholds?, Journal of Dentistry. (2020) 102, 10.1016/j.jdent.2020.103475, 103475. 32961261

[37] Liberato W. F. , de Almeida E. N. , Gallito M. A. , Faria-E-Silva A. L. , Schneider L. F. J. , and Cavalcante L. M. A. , Influence of a Gray Background and the Illuminant on Tooth Shade Selection, The Journal of Prosthetic Dentistry. (2025) 134, no. 4, 1290–1295, 10.1016/j.prosdent.2023.12.005. 38228448

[38] Al Hamad K. Q. , Obaidat I. I. , and Baba N. Z. , The Effect of Ceramic Type and Background Color on Shade Reproducibility of All-Ceramic Restorations, Journal of Prosthodontics. (2020) 29, no. 6, 511–517, 10.1111/jopr.13005. 30537093

